# Pathogenic implications of cerebrospinal fluid barrier pathology in neuromyelitis optica

**DOI:** 10.1007/s00401-017-1682-1

**Published:** 2017-02-09

**Authors:** Yong Guo, Stephen D. Weigand, Bogdan F. Popescu, Vanda A. Lennon, Joseph E. Parisi, Sean J. Pittock, Natalie E. Parks, Stacey L. Clardy, Charles L. Howe, Claudia F. Lucchinetti

**Affiliations:** 1 Department of Neurology, Mayo Clinic, 200 First Street SW, Rochester, MN 55905 USA; 2 Department of Health Sciences Research, Mayo Clinic, Rochester, MN USA; 3 Department of Anatomy and Cell Biology, Cameco MS Neuroscience Research Center, University of Saskatchewan, Saskatoon, SK Canada; 4 Department of Laboratory Medicine and Pathology, Mayo Clinic, Rochester, MN USA; 5 Department of Immunology, Mayo Clinic, Rochester, MN USA; 6 Center for Multiple Sclerosis and Autoimmune Neurology, Mayo Clinic, Rochester, MN USA; 7 Department of Neuroscience, Mayo Clinic, Rochester, MN USA

**Keywords:** Choroid plexus, Leptomeninges, Astrocyte, Complement, Immunopathology, Hydrocephalus

## Abstract

Pathogenic autoantibodies associated with neuromyelitis optica (NMO) induce disease by targeting aquaporin-4 (AQP4) water channels enriched on astrocytic endfeet at blood–brain interfaces. AQP4 is also expressed at cerebrospinal fluid (CSF)–brain interfaces, such as the pial glia limitans and the ependyma and at the choroid plexus blood–CSF barrier. However, little is known regarding pathology at these sites in NMO. Therefore, we evaluated AQP4 expression, microglial reactivity, and complement deposition at pial and ependymal surfaces and in the fourth ventricle choroid plexus in 23 autopsy cases with clinically and/or pathologically confirmed NMO or NMO spectrum disorder. These findings were compared to five cases with multiple sclerosis, five cases of choroid plexus papilloma, and five control cases without central nervous system disease. In the NMO cases, AQP4 immunoreactivity was reduced relative to control levels in the pia (91%; 21/23), ependyma (56%; 9/16), and choroid plexus epithelium (100%; 12/12). AQP4 immunoreactivity was normal in MS cases in these regions. Compared to MS, NMO cases also showed a focal pattern of pial and ependymal complement deposition and more pronounced microglial reactivity. In addition, AQP4 loss, microglial reactivity, and complement deposition colocalized along the pia and ependyma only in NMO cases. Within the choroid plexus, AQP4 loss was coincident with C9neo immunoreactivity on epithelial cell membranes only in NMO cases. These observations demonstrate that NMO immunopathology extends beyond perivascular astrocytic foot processes to include the pia, ependyma, and choroid plexus, suggesting that NMO IgG-induced pathological alterations at CSF–brain and blood–CSF interfaces may contribute to the occurrence of ventriculitis, leptomeningitis, and hydrocephalus observed among NMO patients. Moreover, disruption of the blood–CSF barrier induced by binding of NMO IgG to AQP4 on the basolateral surface of choroid plexus epithelial cells may provide a unique portal for entry of the pathogenic antibody into the central nervous system.

**Electronic supplementary material:**

The online version of this article (doi:10.1007/s00401-017-1682-1) contains supplementary material, which is available to authorized users.

## Introduction

Neuromyelitis optica (NMO) is a disabling inflammatory disorder of the central nervous system (CNS) that is marked by expression of a pathogenic IgG autoantibody directed against the ectodomain of aquaporin-4 (AQP4), the major water channel in the CNS [34, 35]. AQP4 functions to couple bidirectional fast water transport to active ion flux across the plasma membrane, thereby controlling astrocyte homeostasis and CNS osmotic stability [76]. The enrichment of AQP4 on astrocytic endfeet at the blood–brain barrier is consistent with a crucial role in maintaining physiological water balance and with responding to pathological perturbations of this balance associated with ischemia or trauma [75]. Early NMO pathology is characterized by the presence of reactive astrocytes, intramyelinic edema, loss of AQP4 expression, variable perivascular deposition of IgG and complement components, and granulocytic leukocyte infiltration [40, 61]. Advanced lesions demonstrate more profound complement deposition, loss of myelin, and astrocyte destruction. Astrocytic responses in NMO range from sublytic gliosis to overt lysis, and these responses are frequently observed in regions without myelin loss, suggesting that NMO is a primary astrocytopathy associated with secondary demyelination [39]. Such a model for NMO pathogenesis is consistent with observations of water dyshomeostasis [62], lesion reversibility [41, 42, 80], and behavioral sequelae in NMO patients [59].

AQP4 is also expressed at the pial glia limitans, the ependyma, and the choroid plexus [53, 61]. Notably, differential cell–cell junction expression at these barrier sites [78] may provide a unique route for NMO IgG to enter the cerebrospinal fluid (CSF) and access AQP4-expressing targets in the brain parenchyma. Recently, loss of AQP4 expression was observed in cortical layer I in NMO tissue and was associated with cognitive impairment and a corresponding loss of neurons in cortical layer II [65]. This finding suggests that subpial AQP4 was targeted by NMO IgG and indicates that astrocytopathy outside of the typical periventricular lesion may have profound pathogenic and neurologic consequences. However, at present, little is known about the pathology of CSF–brain and blood–CSF barriers in NMO patients. Therefore, in this study, we analyzed astrocyte and microglia reactivity, AQP4 expression level, and complement deposition at these interfaces.

## Materials and methods

### Study design and series

This study was approved by the Institutional Review Board of Mayo Clinic, Rochester, MN (IRB 2067-99). Inclusion criteria were (i) clinical and pathological diagnosis of NMO or NMOSD; (ii) sufficient archival tissue for pathological analysis; and (iii) no evidence of alternative diagnosis. Twenty-three autopsy cases met the inclusion criteria (315 total tissue blocks) (Supplemental Figure 1). Table 1 provides demographics for the patient cohort. As controls, we included five multiple sclerosis (MS) cases (79 blocks; 4 relapsing remitting MS, 1 secondary progressive MS) and five control cases without known CNS disease (45 blocks). As additional controls for choroid plexus disease, we included five hydrocephalus cases (five blocks) and five papilloma cases (five blocks). We analyzed supratentorial brain (including optic nerve), brain stem, cerebellum, and spinal cord for histopathological abnormalities in the pial surface, ependyma, and choroid plexus. To reduce anatomical variability in the choroid plexus analysis, we specifically assessed the choroid plexus in the fourth ventricle at the level of the medulla.

**Table 1:** Demographic and clinical summaries

Characteristic	Summary
Number of subjects	23
Sex, male:female	2:21
Age at symptom onset, years	49 (12–73)
Diagnosis, *n* (%)	
NMO	18 (78%)
NMO spectrum disorder	5 (22%)
AQP4-IgG serostatus, positive:negative^a^	9:0
Number of clinical attacks	3 (2–7)
Disease duration, months	36 (8–240)
Age at death, years	52 (16–80)

Unless otherwise indicated values shown are median (range). Note: age of symptom onset and disease duration missing for one patient; number of clinical attacks missing for two patients; AQP4-IgG serology missing for 14 patients

^a^Sera available for testing in nine patients. Other subjects either lacked sera or preceded the availability of serological testing

### Neuropathological evaluation

Formalin-fixed paraffin-embedded 5 µm thick sections were stained with hematoxylin and eosin (H&E), Luxol fast blue, and periodic acid Schiff (LFB/PAS), and modified Bielschowsky silver. Immunohistochemistry was performed with the avidin–biotin-complex method as previously reported [40], using primary antibodies against glial fibrillary acidic protein (GFAP, 1:100, DAKO, Denmark), neurofilament (1:800, steam antigen retrieval with citric acid buffer pH 6.0, DAKO, Denmark), AQP1 (1:250; Santa Cruz), AQP4 (1:250, Sigma-Aldrich, USA), myelin proteolipid protein (PLP, 1:500, Serotec, Oxford, UK), KiM1P (pan-macrophage marker, 1:5000, from Dr. Wolfgang Bruck, Germany), complement C9 neo-antigen (C9neo, monoclonal B7 and polyclonal, 1:200, from Professor Paul Morgan, Cardiff, UK), and human IgG gamma chain (1:200, DAKO, Denmark).

We systematically analyzed the pial surface, ependyma, and choroid plexus for histopathological alterations, and assessed AQP4 immunoreactivity, the pattern of macrophage/microglial reaction, and the presence of C9neo deposition. The patterns observed in NMO tissue were compared to the corresponding anatomical areas in MS and controls. For quantitation of AQP4 loss, the entire pia available on the tissue block was assessed for AQP4 immunoreactivity. The pattern of AQP4 reactivity was defined as focal when loss was restricted to a single high-power field at 40X, or diffuse when extending beyond one such field. Because of variation in the extent of the diffuse involvement and to help differentiate between the situation, where diffuse loss encompassed the bulk of the pial surface versus when the diffuse loss was relatively isolated, we categorized the diffuse loss as occupying <25, 25–50, 50–75, or >75% of the total length of pia available for analysis.

## Results

### Pial glia limitans

**Fig. 1:**
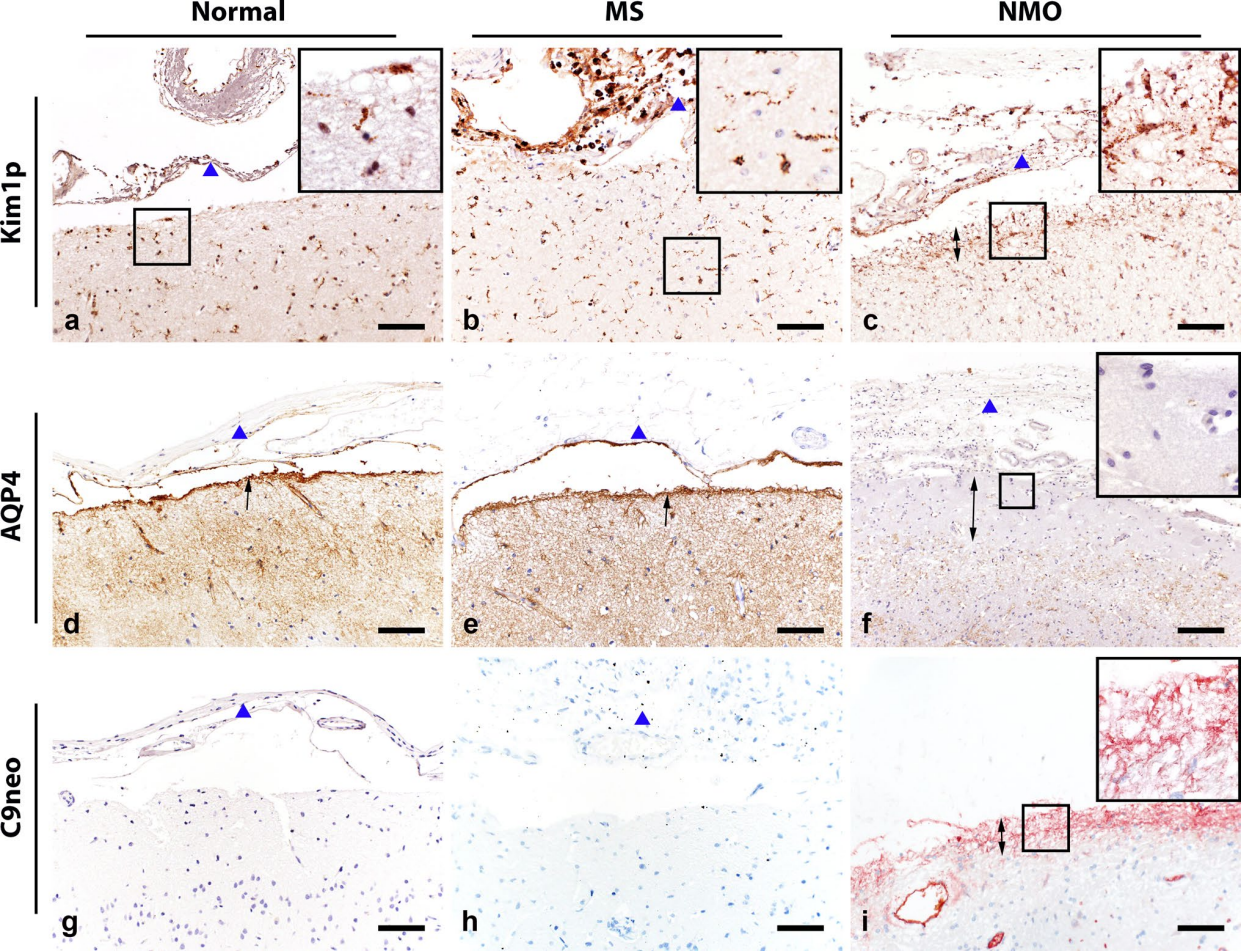
Pial pathology in normal, MS, and NMO tissues. Supratentorial brain tissue from neurologically normal controls (**a**, **d**, **g**), MS (**b**, **e**, **h**), and NMO (**c**, **f**, **i**). Microglial reactivity is minimal at the pial interface in normal control tissue (**a**), prominent in MS meninges, with diffuse extension into the cortical parenchyma (**b**), and concentrated in the glial limitans and in the upper molecular layer (between *arrowheads*) in NMO (**c**). AQP4 immunoreactivity is abundant at the glia limitans in normal control (**d**) and MS (**e**) (*arrows*), but lost or markedly reduced along the cortical surface in NMO (**f**; between *arrowheads*). Complement C9neo immunoreactivity is not detected at the pia in normal control (**g**) or MS (**h**), but concentrated at the pia in NMO (**i**; between *arrowheads*). Immunohistochemistry for KiM1P (**a**–**c**), AQP4 (**d**–**f**), and C9neo (**g**–**i**). Meninges marked by *block up pointing triangle*. *Scale bars* 100 µm

At the pial surface of the cerebral cortex, normal control tissue showed minimal microglial reactivity (KiM1P expression) (Fig. 1a), abundant AQP4 immunoreactivity (Fig. 1d), and no evidence of complement C9neo deposition (Fig. 1g). Widespread microglial reactivity, in both the meninges and subpial cortex, was observed in MS tissue (Fig. 1b), but AQP4 immunoreactivity was normal (Fig. 1e) and C9neo deposition was not detected (Fig. 1h). In contrast, in NMO tissue, there was accumulation of reactive microglia (Fig. 1c), near complete loss of AQP4 immunoreactivity at the CSF–brain interface (Fig. 1f) and deposition of C9neo in focal regions of the pial glia limitans (Fig. 1i).

**Fig. 2:**
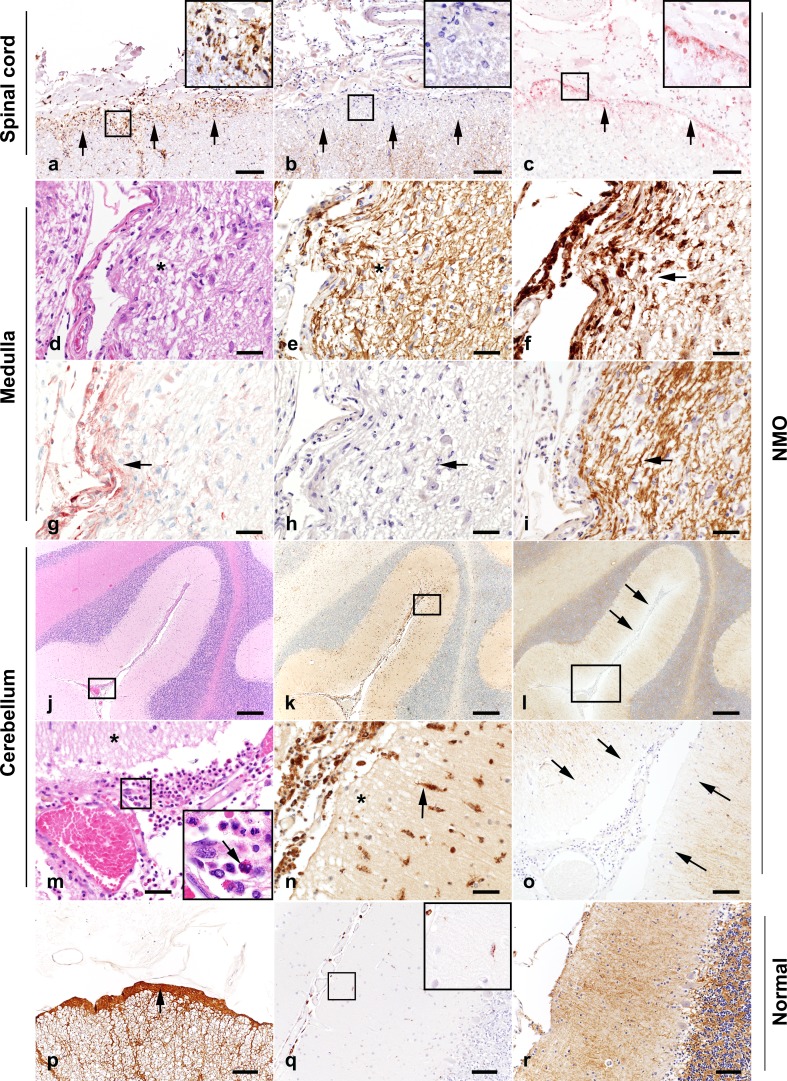
Pial pathology in NMO. Spinal cord (**a**–**c**): Microglial reactivity (*arrows*) is concentrated at the pial glia limitans (**a**), colocalizing to a region of AQP4 loss (**b**), and C9neo deposition (**c**), on consecutive sections. Medulla oblongata: (**d**–**i**; consecutive sections). The subpial parenchyma exhibits mild vacuolation consistent with edema (**d**; H&E), dystrophic astrocytes (**e**), microglial reactivity (**f**), C9neo deposition (**g**), loss of AQP4 immunoreactivity (**h**), and preserved myelin (**i**). Cerebellum (**j**–**o**): **j** Cerebellar meninges contain infiltrating eosinophils and neutrophils (H&E; enlarged in **m**) and subpial vacuolation (* in **m**). Reactive microglia are distributed in the subpial molecular layer (**k**; enlarged in **n**). Subpial AQP4 immunoreactivity is lost (**l**; enlarged in **o**). Normal control: AQP4 immunoreactivity is abundant at the surface of the spinal cord (**p**) and cerebellum (**r**). Microglial reactivity is negligible in the normal cerebellum (**q**). Immunohistochemistry for KiM1P (**a**–**c**, **f**; **k**, **n**, **q**); AQP4 (**b**, **h**, **l**, **o**, **p**, **r**); C9neo (**c**, **g**); GFAP (**e**); PLP (**i**). *Scale bars* in **a**, **b**, **c**, **p**, **q**, and **r** = 100 µm. *Scale bars* in **d**–**i**, **m**-**o** = 50 µm. *Scale bars* in **j**, **k**, **l** = 200 µm

In NMO spinal cord, foci of reactive microglia (Fig. 2a), AQP4 loss (Fig. 2b; compare to normal spinal cord in Fig. 2p), and C9neo deposition (Fig. 2c) were observed at the pial glia limitans. At the pial surface of the medulla oblongata, dystrophic astrocytes (Fig. 2d, e), reactive microglia (Fig. 2f), C9neo deposition (Fig. 2g), and AQP4 loss (Fig. 2h) were observed within the context of preserved myelin (Fig. 2i). At the pial surface of the cerebellum, meningeal eosinophils and neutrophils (Fig. 2j, m) were found in proximity to regions containing reactive microglia (Fig. 2k, n; compare to normal cerebellum in Fig. 2q) and decreased or absent AQP4 immunoreactivity (Fig. 2l, o; compare to normal cerebellum in Fig. 2r). At the cerebellar pia, there was a mild loss of astrocytic processes (Supplementary Figure 2a, 2b), compared to normal cerebellum (Supplementary Figure 2c, 2d).

**Fig. 3:**
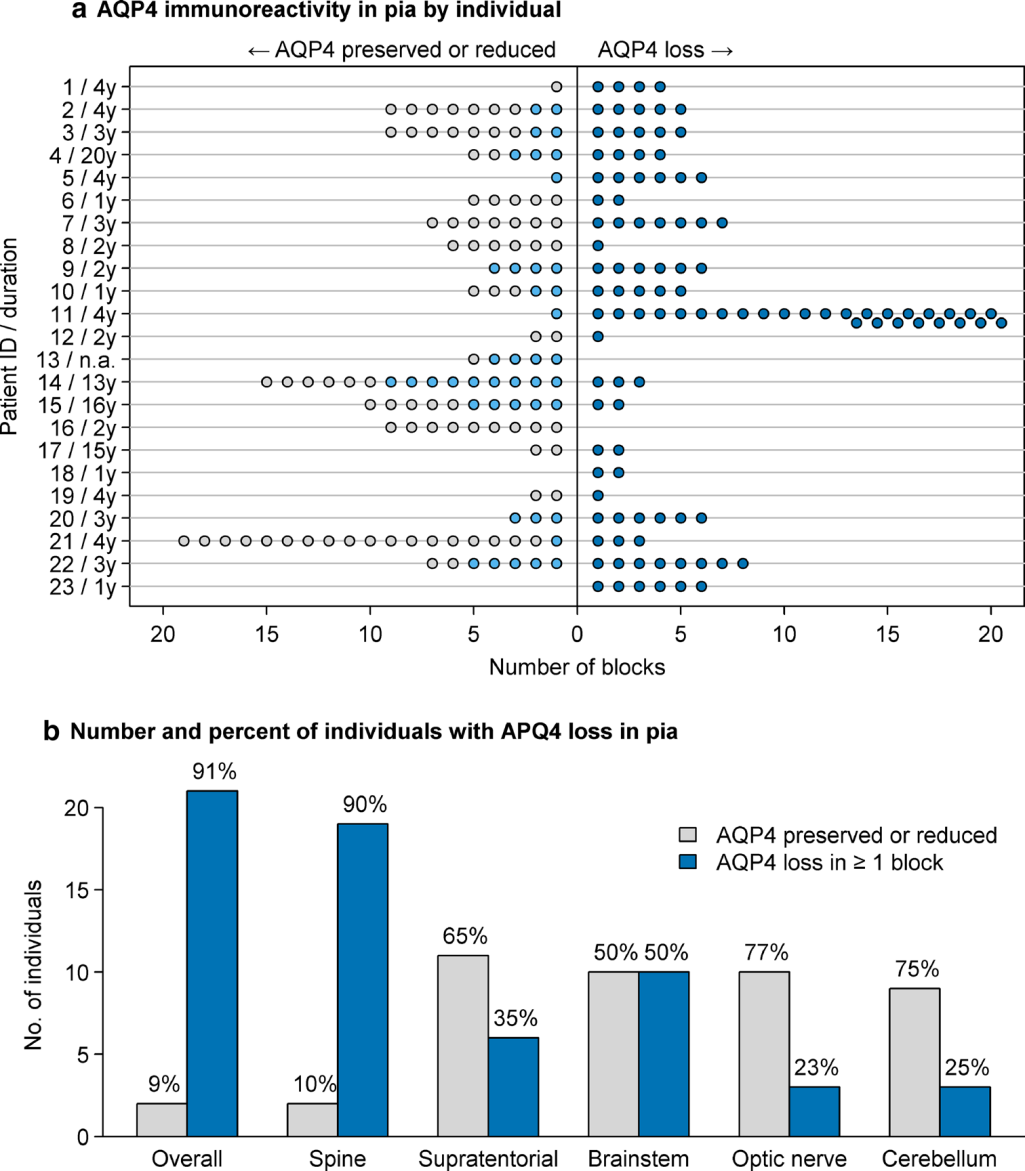
Quantification of pial AQP4 immunoreactivity. AQP4 immunoreactivity in the pia was assessed in multiple blocks from individual patients (**a**). A range of AQP4 expression was observed. *Dark blue circles* enumerate blocks with AQP4 loss; *light blue circles* represent *blocks* that showed reduced AQP4 but not complete loss, and *gray circles* are *blocks* with normal or increased AQP4. The number of NMO cases exhibiting AQP4 loss versus AQP4 preservation in the pia in at least one block was determined across anatomical regions and expressed as percentages (**b**)

**Fig. 4:**
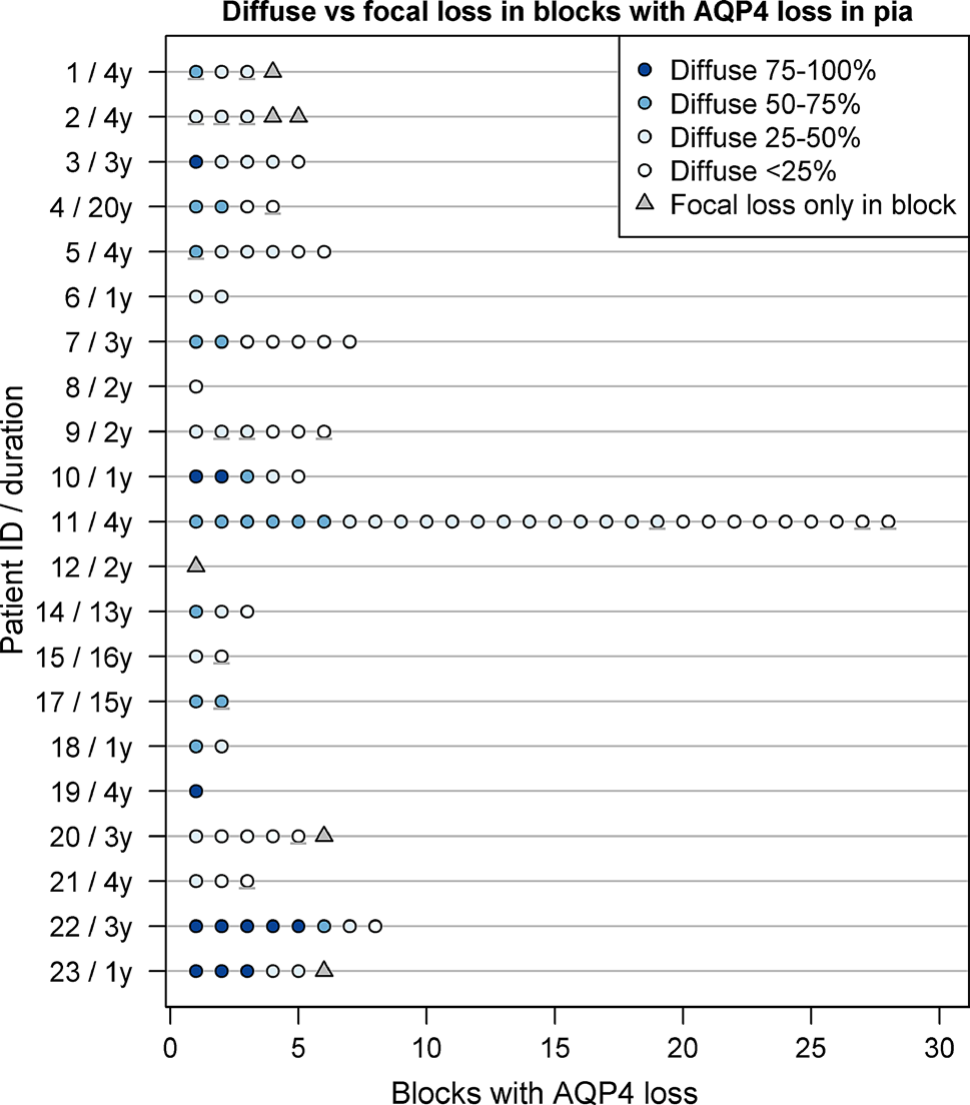
Pattern of pial AQP4 immunoreactivity loss in individual NMO patients. Among 21 NMO cases (107 blocks), the pattern of pial AQP4 loss was characterized as focal or diffuse. Subjects exhibiting diffuse AQP4 loss were further graded based on the percentage of pial involvement ranging from <25%; 25–50%; 50–75%, and >75% loss. *Each symbol* represents one block; *symbol shape* represents pattern of loss; and *color* indicates percentage of pia demonstrating diffuse AQP4 loss. Blocks exhibiting both diffuse and focal patterns are indicated with a *gray line* under the *color*-coded diffuse pattern *symbol*. Patient identifiers and disease duration are shown on the *y*-axis

In 21 of 23 NMO cases (91%), AQP4 immunoreactivity was lost and/or decreased at the pia across multiple blocks examined from a given patient (Fig. 3a). All CNS regions exhibited AQP4 loss at the pia, most frequently in spinal cord (Fig. 3b, Supplementary Figure 3). Reduced AQP4 immunoreactivity occurred in both a diffuse and focal pattern (Fig. 4), with diffuse loss ranging from less than 25% of the sampled pia, to more than 75% of the region characterized by AQP4 loss.

The majority (82%) of NMO cases exhibited reactive microglia that were increased in both size and number (Supplementary Figure 4a, 4b). In cerebral cortex and spinal cord, the microglial reaction was preferentially distributed along the pial glia limitans, but in cerebellum, reactive cells were distributed at both the pial surface and in the subpial parenchyma. With regard to complement C9neo staining, 81% of NMO cases exhibited complement deposits at the pial surface (Supplementary Figure 5a, 5b; Table 2).

**Table 2:** Comparison of pathology at CSF–brain and blood–CSF interfaces

	NMO	MS	Normal
Pial surface			
C9neo deposition	Yes (17/21)	Yes (2/5)	No
Microglial reaction			
Localized to pia	Yes (18/22)	Yes	No
Extended into cortex	Rare	Yes	No
Associated with CDM	No	No	No
AQP4 loss	Yes (21/23)	No (increased)	No
Ependyma			
C9neo deposition	Yes (6/16)	Yes (1/5)	No
Microglial reaction	Yes (16/16)	Scattered (2/5)	No
AQP4 loss	Yes (9/16)	No (increased)	No
Ependymal discontinuity	Yes (16/16)	Yes (5/5)	Rare
Choroid plexus^a^			
C9neo deposition			
On epithelial membrane	Yes (4/10)	No	No
In stroma	Yes	Yes	Yes (rare)
Microglial reaction	Moderate	Mild	Minimal
AQP4 loss	Yes (12/12)	No (increased)	No

*CDM* cortical demyelination

^a^Epithelial cells in the normal choroid plexus of the fourth ventricle express AQP4. In NMO, AQP4 loss was reflected by a decrease in the number of positive cells and by reduced AQP4 staining intensity on positive cells

**Fig. 5:**
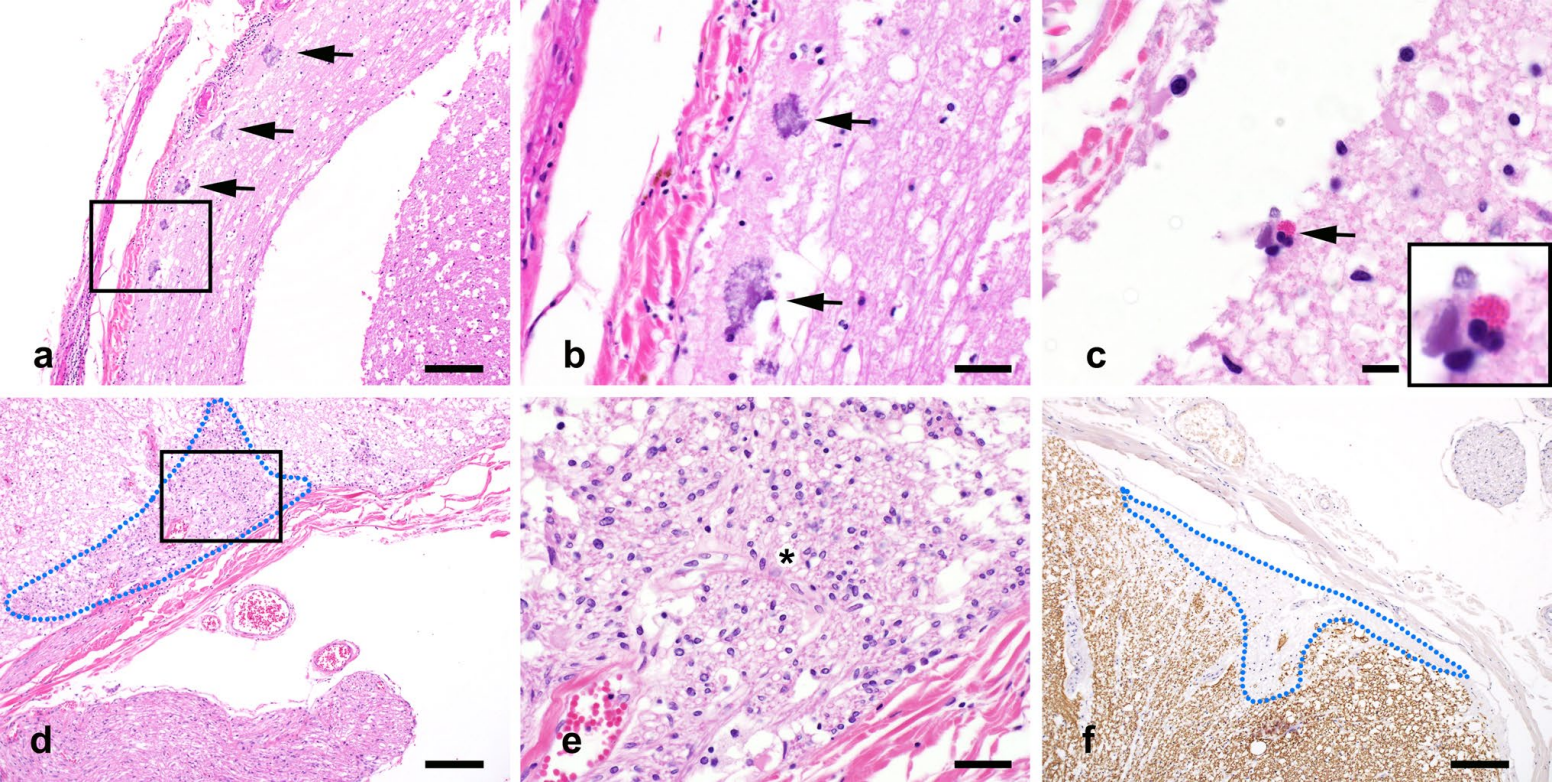
Spectrum of subpial histopathological alterations in NMO. Calcifications are observed in the spinal pial glia limitans (**a**; *arrows*; enlarged in **b**; H&E). An eosinophil is present in the pial glia limitans immediately below the spinal leptomeninges (**c**; *arrow*; H&E). Invading Schwann cells (*circled with blue dotted line*) are found in the spinal cord at the dorsal root entry zone (**d**; enlarged in **e**; H&E). Focal subpial myelin loss (*circled with blue dotted line*) is observed in the spinal cord (**f**; PLP). *Scale bars* in **a**, **d**, and **f** = 200 µm. *Scale bars* in **b** and **e** = 50 µm. *Scale bars* in **c** = 10 µm

Colocalization of microglial activation, AQP4 loss, and C9neo deposition at the pia was only observed in NMO tissue and never in controls or MS samples. Furthermore, all 23 NMO cases exhibited pial and subpial tissue vacuolation characterized by enlarged spaces between astrocytic processes (Fig. 2d, n). In some regions, this was coincident with dystrophic or hypertrophic astrocytes (Fig. 2e). Subpial calcifications (Fig. 5a, b) and infiltrating inflammatory cells (Fig. 5c) were also observed at the pial glia limitans in both NMO brain and spinal cord. Focal subpial myelin loss (Fig. 5f) was evident in the brain stem and spinal cord white matter, but not in the cortex. In some regions of myelin loss, axons were remyelinated by CNS-infiltrating Schwann cells (Fig. 5d, e).

### Ependyma

**Fig. 6:**
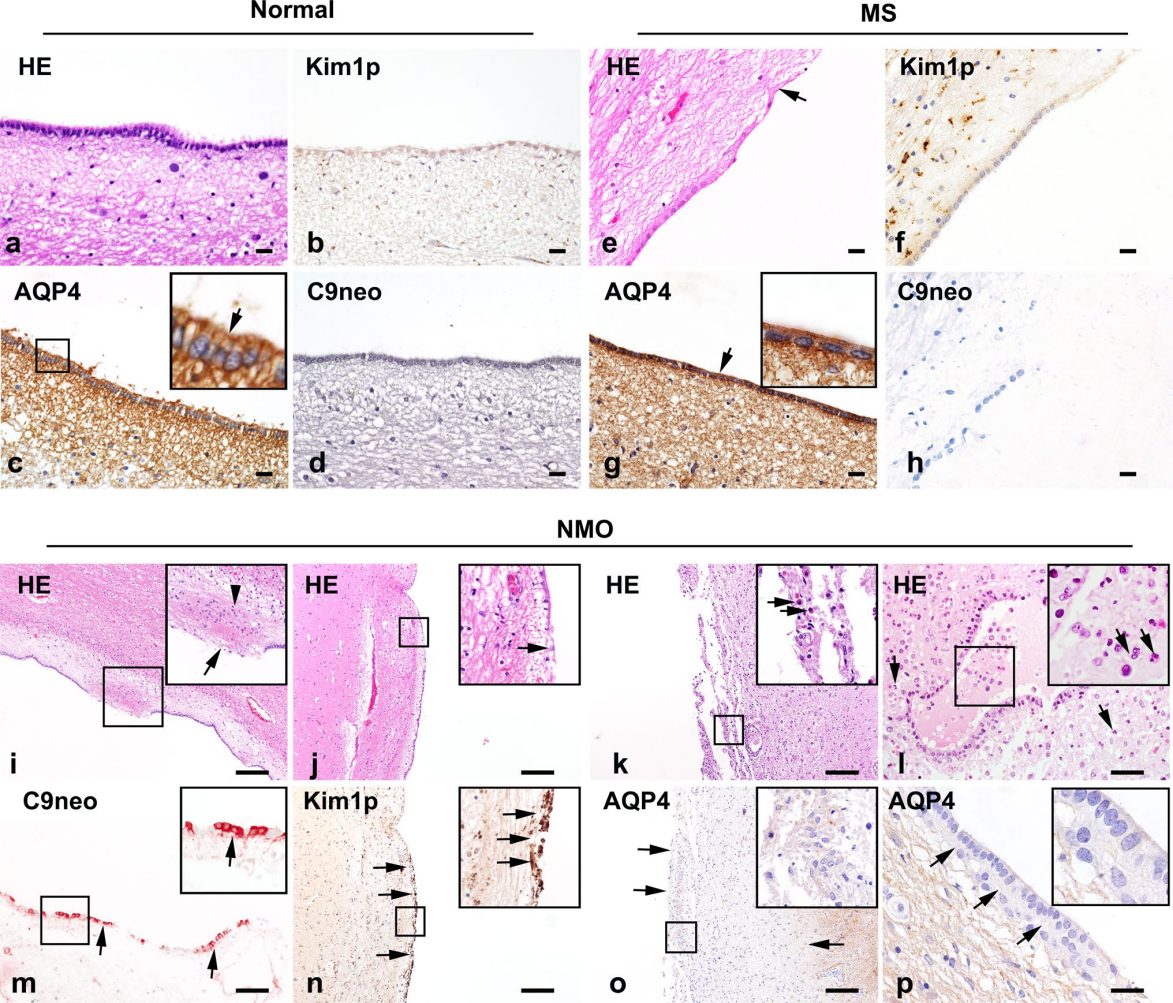
Ependymal pathology in normal, MS, and NMO tissues. Normal control: Ependymocytes show ciliated simple columnar morphology lining the ventricle (**a**). Minimal microglial reactivity is present (**b**), AQP4 immunoreactivity is abundant on ependymocytes and in the subependymal parenchyma (**c**), and C9neo is not detected (**d**). MS: Focal ependymal loss is present in MS (**e**; *arrow*). Reactive microglia are found in the subependymal parenchyma but not in the ependymal layer (**f**); AQP4 immunoreactivity is increased at the ependyma (**g**; *arrow*), but C9neo is not present (**h**). NMO: Ependymocytes are lost (*arrow*) and a subependymal glial nodule (*arrowhead*) bulges into the ventricle (**i**). Discontinuous atrophic ependymal cells (*arrow*) are flattened along the ventricle wall (**j**). Infiltrating eosinophils (*arrows*) are present beneath a disorganized ependymal layer (**k**). Abundant granulocytes are found on the CSF face of preserved ependyma and in subependymal parenchyma (**l**). C9neo immunoreactivity is observed on ependymocytes (**m**). A consecutive section to (**j**) demonstrates microglial reactivity (*arrows*) at the ependymal layer (**n**). AQP4 loss (**o**) is extensive in both the ependyma and subependymal regions of a section consecutive to (**k**). Focal loss of ependymal AQP4 immunoreactivity is observed with preservation of subependymal AQP4 (**p**). *Scale bars* in **a**–**h** = 20 µm. *Scale bars* in **i**–**k**, **n,** and **o** = 200 µm. *Scale bars* in **l**, **m**, and **p** = 100 µm. Immunohistochemistry for KiM1P (**b**, **f**, **n**); AQP4 (**c**, **g**, **o**, **p**); C9neo (**d**, **h**, **m**); and H&E (**a**, **e**, **i**–**l**)

The ependyma is formed by a continuous layer of ciliated ependymocytes arranged in a simple columnar organization that separates the ventricle from the parenchyma (Fig. 6a). In normal control tissue ependymal cells expressed abundant AQP4 enriched on the basolateral membrane (Fig. 6c) in the absence of reactive microglia (Fig. 6b) or C9neo deposition (Fig. 6d). In MS tissue, focal discontinuities in the ependymal layer were occasionally observed (Fig. 6e) and the ependymocytes present exhibited increased AQP4 immunoreactivity (Fig. 6g). While subependymal reactive microglia (Fig. 6f) were variably found in MS tissues, there was no evidence of C9neo deposition in or near the ependyma (Fig. 6h). In contrast, all 16 NMO cases with ependymal tissue available for analysis exhibited loss of ependymocytes and these discontinuities were typically associated with subependymal gliosis and glial nodules extending into the ventricle (Fig. 6i). Where the ependymal layer remained intact, the cells were frequently characterized by an atrophic, flattened morphology, and irregular organization (Fig. 6j), including the presence of periventricular ependymal rosettes. This disorganized ependyma was also associated with variable eosinophilic infiltration (Fig. 6k) and the presence of granulocytes along the CSF face of the layer and in the subependymal parenchyma (Fig. 6l).

**Fig. 7:**
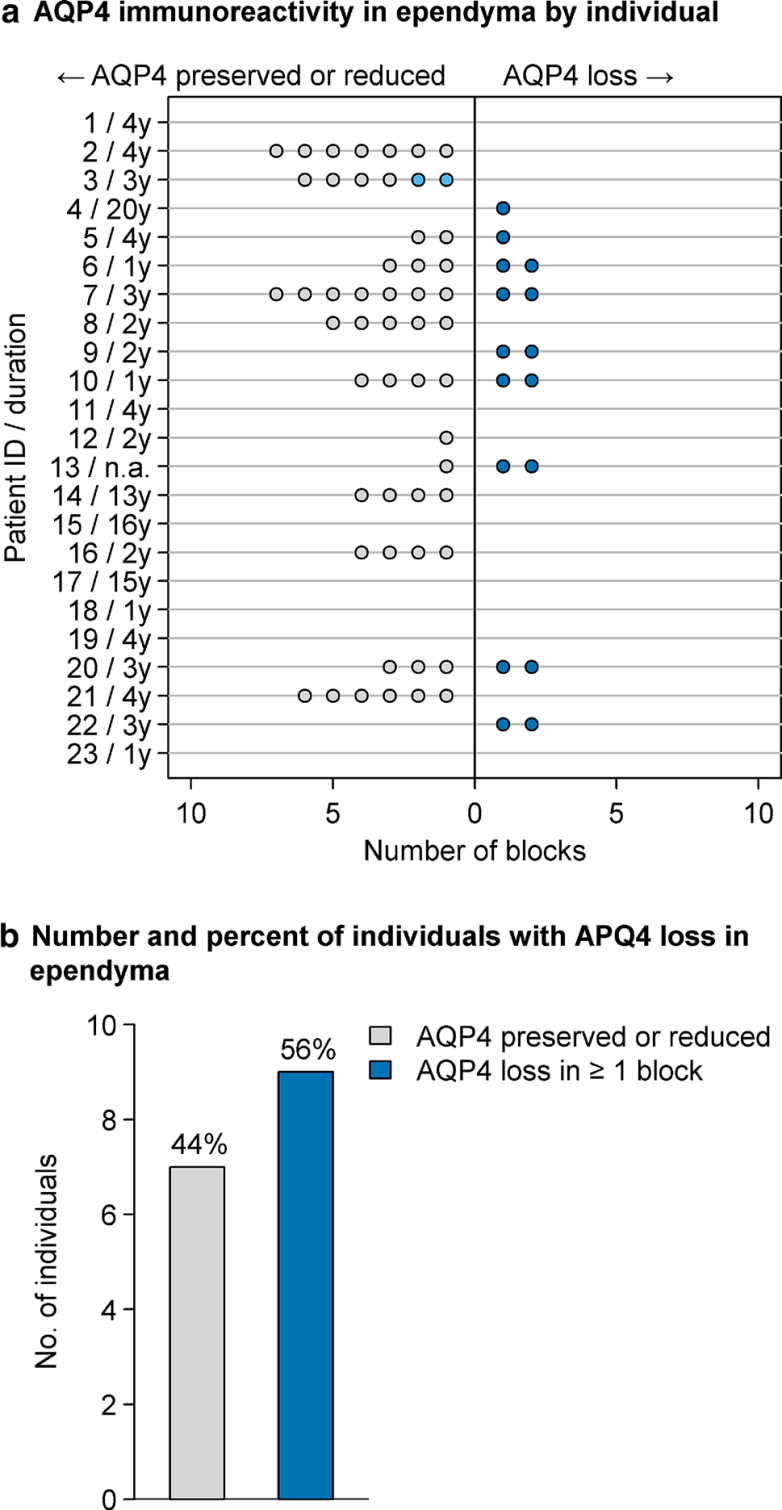
Quantification of ependymal AQP4 immunoreactivity in NMO. AQP4 immunoreactivity in the ependyma was assessed in multiple blocks from individual patients (**a**). A range of AQP4 expression was observed. *Dark blue circles* enumerate blocks with AQP4 loss; *light blue circles* represent blocks that showed reduced AQP4 but not complete loss; and *gray circles* are blocks with normal or increased AQP4. Patient identifiers and disease duration are shown on the *y*-axis. The number of NMO cases exhibiting AQP4 loss versus preservation in the ependyma in at least one block was determined and expressed as a percentage (**b**)

AQP4 immunoreactivity was lost from preserved ependymocytes across multiple blocks among 56% of 16 NMO cases with available tissue (Fig. 7a, b), often extending into the subependymal parenchyma (Fig. 6o). A combination of subependymal astrocyte reactivity with variable astrocyte loss was evident in these regions (Supplementary Figure 6). In some cases, AQP4 was lost from intact ependymocytes but preserved in the parenchyma, suggesting early involvement at the CSF interface (Fig. 6p). All but one of the 16 NMO cases (94%) also exhibited reactive microglia in the ependymal layer (Fig. 6n and Supplementary Figure 7) and about a third (38%) of cases showed C9neo deposition preferentially localized to the ependymocytes (Fig. 6m and Supplementary Figure 8).

### Choroid plexus

**Fig. 8:**
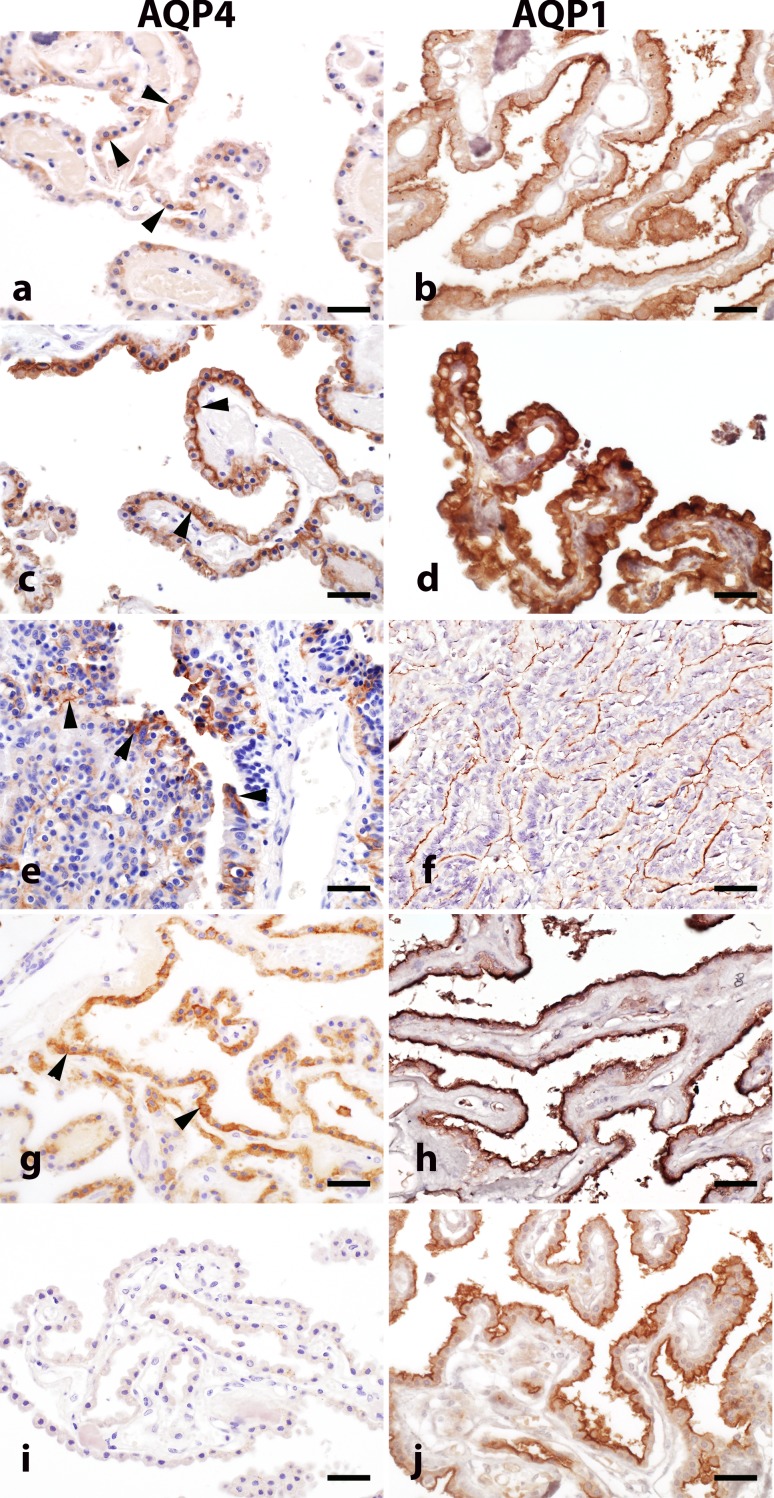
Patterns of AQP4 and AQP1 expression in the choroid plexus. Normal choroid plexus epithelial cells exhibit variable AQP4 immunoreactivity in the basolateral membrane and cytoplasm (**a**; *arrowheads highlight positive cells*), whereas AQP1 is distributed uniformly at the apical membrane in normal choroid plexus epithelial cells (**b**). In hydrocephalus, both AQP4 (**c**) and AQP1 (**d**) immunoreactivity are increased throughout the choroid epithelium. AQP4 immunoreactivity is focally increased in papilloma (**e**; *arrowheads*), while AQP1 expression (**f**) is more variable and abnormal. In multiple sclerosis, AQP4 immunoreactivity in the choroid plexus is either normal or increased (**g**; *arrowheads*) with more pronounced AQP1 immunoreactivity (**h**). In NMO, AQP4 immunoreactivity is greatly reduced or entirely absent in the choroid plexus (**i**); however, AQP1 immunoreactivity is normal (**j**). *Scale bars* 20 µm. Immunohistochemistry for AQP4 (**a**, **c**, **e**, **g**, **i**) and AQP1 (**b**, **d**, **f**, **h**, **j**)

In normal controls, approximately 40% of choroid plexus epithelial cells demonstrate AQP4 immunoreactivity in the cytoplasm and at the basolateral membrane (Fig. 8a, arrows). In general, AQP4 expression was more abundant in choroid plexus adjacent to the wall of the fourth ventricle. AQP1 immunoreactivity was more uniform in normal choroid plexus and predominantly localized to the epithelial cell apical membrane (Fig. 8b). Compared to normal control tissue, the choroid plexus in hydrocephalus cases exhibited more pronounced basolateral AQP4 immunoreactivity (Fig. 8c) and increased AQP1 immunoreactivity in the cytoplasm and at the apical plasma membrane (Fig. 8d). All five papilloma cases showed focal clusters of tumor cells with increased AQP4 immunoreactivity in both the membrane and cytoplasm, without apparent polarization (Fig. 8e). AQP1 immunoreactivity remained polarized to the apical membrane in papilloma tissue, but the staining pattern was heterogeneous (Fig. 8f). AQP4 immunoreactivity was normal or increased in MS choroid plexus (Fig. 8g), and apical AQP1 immunoreactivity was increased in some cases (Fig. 8h). In contrast, in all 12 NMO cases with choroid tissue available, choroid plexus epithelial cells demonstrated a near complete loss of AQP4 immunoreactivity (Fig. 8i), whereas AQP1 immunoreactivity was preserved (Fig. 8j).

**Fig. 9:**
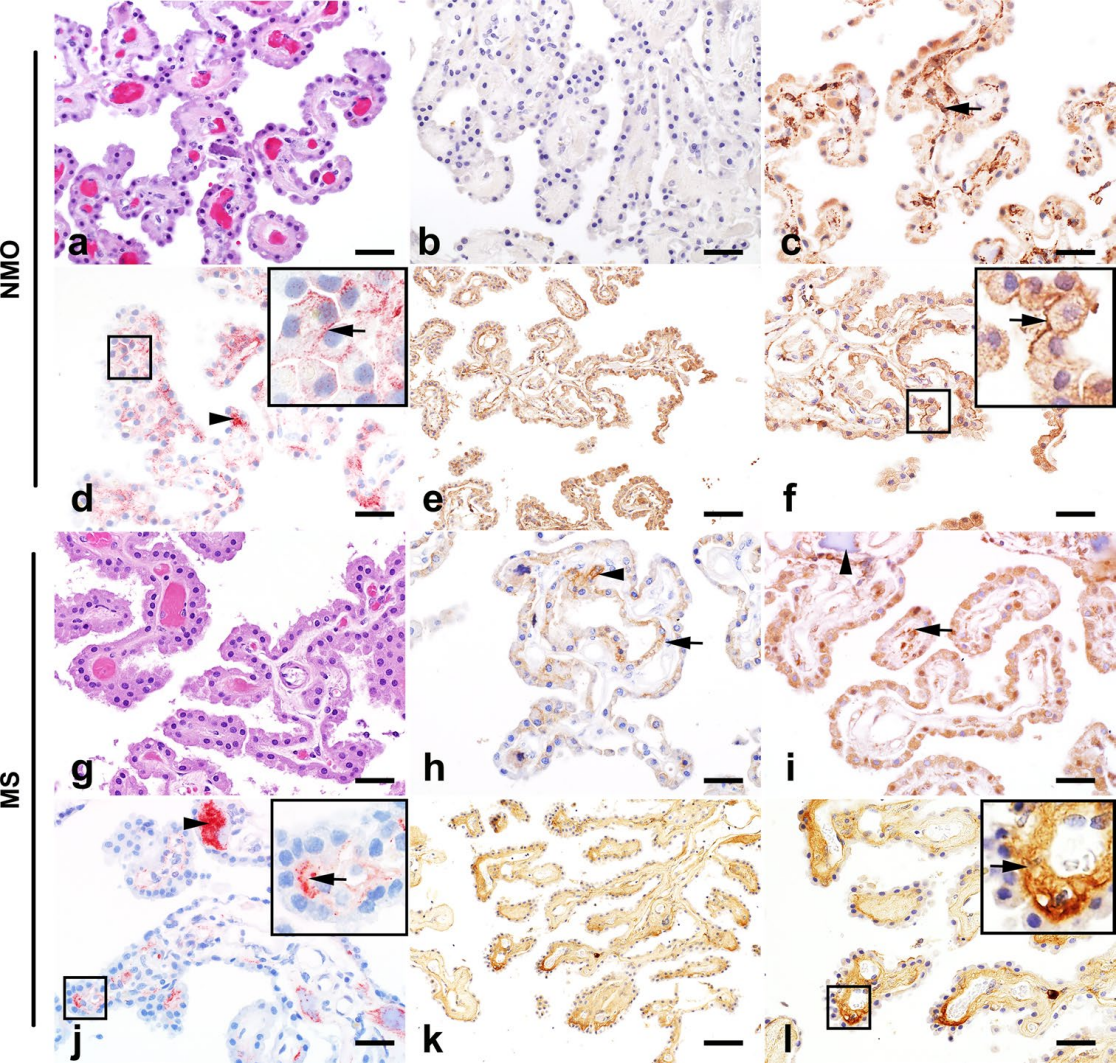
Choroid plexus pathology in NMO and MS. NMO: Intact choroid plexus (**a**; H&E) exhibits extensive AQP4 loss (**b**) and increased macrophage density (**c**). C9neo deposition is observed in stromal concretions (**d**; *arrowhead*) but also on choroid epithelial cell membranes (**d** *inset*; *arrow*). IgG immunoreactivity is also observed in the choroid plexus (**e**) with intense IgG immunoreactivity on the membrane of choroid plexus epithelial cells (**f**; *inset*). MS: Choroid plexus (**g**; H&E) shows preserved AQP4 (**h**). Scattered macrophages are found in the stroma (**i**; *arrows*), as well as calcifications (**i**; *arrowhead*). C9neo deposition is present in stromal concretions (**j**; *arrowhead*) and in the perivascular basement membrane (**j** *inset*; *arrow*) but not on choroid epithelial cell membranes. Diffuse IgG immunoreactivity is observed in the choroid plexus (**k**), predominantly restricted to the stroma and perivascular region (**l**; *arrow*; *inset*). *Scale bars* in **a**–**d**, **f**, **g**–**j,** and **l** = 50 µm. *Scale bars* in **e** and **k** = 100 µm. Immunohistochemistry for AQP4 (**b**, **h**); KiM1P (**c**, **i**); C9neo (**d**, **j**); and IgG (**e**, **f**, **k**, **l**)

Further analysis of NMO cases confirmed that while the gross structure of choroid plexus did not appear disrupted (Fig. 9a), AQP4 immunoreactivity in the choroid epithelial cells was reduced or lost from the basolateral membrane (Fig. 9b). In contrast, the intact choroid plexus in MS cases (Fig. 9g) exhibited normal or increased AQP4 immunoreactivity (Fig. 9h). The number and size of stromal KiM1P-positive cells were increased in 86% of NMO cases (Fig. 9c), while only scattered KiM1P-positive macrophages/microglia were observed in the MS cases (Fig. 9i). C9neo deposition was observed in perivascular regions of the plexus stroma and in stromal concretions in both NMO (arrowhead in Fig. 9d) and MS cases (arrowhead in Fig. 9j). However, extensive C9neo immunoreactivity was exclusively found on the membrane of choroid plexus epithelial cells in 40% of NMO cases (Fig. 9d), a pattern that was not observed in any of the MS cases. Membrane Ig deposition was present on choroid epithelial cells in NMO (Fig. 9e, f), but restricted to the choroid stroma in MS (Fig. 9k, l).

## Discussion

This study provides the first evidence for involvement of CSF–brain and blood–CSF interfaces at the pial glia limitans, the ependymal surface, and the choroid plexus in the histopathology of NMO (Table 2). At the pial glia limitans, AQP4 loss was colocalized with dystrophic astrocytes, microglial reactivity, and C9neo deposition. AQP4 loss at the pial surface also colocalized with meningeal infiltration of eosinophils and neutrophils. Likewise, the ependymal lining of ventricles in NMO tissue exhibited loss of ependymocytes and structural disorganization associated with the presence of subependymal reactive astrocytes, loss of AQP4 expression at both the ependymal surface and in the parenchyma, microglial reactivity, C9neo deposition, and granulocytic infiltrates at the CSF face of the ependymal layer. Finally, the choroid plexus in NMO tissues exhibited profound loss of AQP4 expression, an increase in number of stromal macrophages, and deposition of immunoglobulins and complement activation factors on choroid epithelial cell membranes. These changes were never observed in control tissues. The specificity of AQP4 loss in the choroid plexus in NMO cases was confirmed by the coincident retention of normal levels of AQP1 immunoreactivity. While MS cases did exhibit microglial reactivity and some evidence of stromal complement deposition, the pattern and extent of pathology were markedly different from NMO, indicating a unique pathology involving CSF interfaces in NMO that mirrors the pathology at blood–brain interfaces. Notably, while the glia limitans superficialis and the glia limitans perivascularis share a primary involvement of AQP4-expressing astrocytic foot processes, the ependymal and choroid plexus barriers are comprised of non-astrocytic cells expressing AQP4. Thus, while NMO is certainly a primary astrocytopathy, the disease also has an important component of an “AQP4-opathy” that involves antibody-mediated injury to other CNS cell types that express the water channel. As with our previous report demonstrating that sarcolemmal AQP4 is a target of IgG in patients with NMO [19], these observations further expand the cellular repertoire of NMO IgG targets beyond the archetypal astrocyte.

Ependymoglial lineage cells exhibit apical-basal polarization and the expression of basolateral membrane specializations that facilitate contact with mesenchymal borders [10]. These specializations anchor the basolateral membrane of ependymoglial cells to the basal lamina of mesenchymal cells via interactions between laminin, α-dystroglycan, and β-dystroglycan [73]. Thus, astrocytic endfeet interact with capillary endothelial cell and meningeal fibroblast basement membranes to form the blood–brain barrier and the CSF–brain barrier, respectively [69], and choroid plexus epithelial cells interact with capillary endothelial cell basement membranes to form the blood–CSF barrier [28, 37]. These physical mesenchymal–ependymoglial barriers are vital to the maintenance of the unique CNS interstitial fluid composition required for normal neuronal function. Of note, AQP4 is a component of the dystrophin-associated protein complex and is, therefore, polarized to the basolateral membrane of ependymoglial cells, bringing the water channel into proximity with the mesenchymal borders [50]. Indeed, this localization is fundamental to water, ion, and protein exchange between the blood, the brain parenchyma, and the CSF [1].

The blood–brain barrier is formed at tight junctions between capillary endothelial cells within the brain parenchyma, preventing diffusion of blood-borne ions and proteins into the perivascular stroma. The endfeet of perivascular astrocytes, along with accessory cells, such as pericytes, provides a second layer of control over solute flow from blood to parenchyma, resulting in a two-step process of facilitated movement across the endothelial cell barrier and then across the astrocyte barrier [69]. In contrast, the blood–CSF barrier at the choroid plexus is physically manifested by the apical tight junctions found between choroid plexus epithelial cells. The endothelial cells at this interface are fenestrated and lack tight junctions, permitting diffusion of ions and proteins into the choroid stroma [74]. Pathogenically, this difference has a profound consequence for accessibility of NMO IgG to AQP4. At the blood–brain barrier, circulating NMO IgG has limited access to AQP4 on perivascular astrocytic endfeet. However, at the blood–CSF barrier, NMO IgG could readily enter the perivascular space and encounter AQP4 on the basolateral surface of the choroid epithelial cells.

Robust AQP4 expression on the basolateral surface of choroid plexus epithelial cells in normal tissue coupled to the presence of immunoglobulin immunoreactivity and the nearly complete loss of AQP4 expression in these cells in NMO tissue indicates that the blood–CSF barrier may be a primary site for entry of NMO IgG into the CNS. In fact, immunoglobulins have been detected within the choroid plexus stroma in healthy normal controls [52], as well as in both neurological and non-neurological disease conditions, such as Alzheimer disease, systemic lupus erythematosus, and others [3, 4, 9, 13, 17, 46, 55, 56, 66, 67, 81]. Of note, we also detected robust immunoreactivity for C9neo at the blood–CSF barrier in NMO tissues, with a pattern that suggested deposition along the choroid plexus epithelial cell membrane. Complement factors, ranging from C1q through to C9neo, are also observed in the choroid plexus in multiple conditions, including MS and Alzheimer disease [22, 48, 54–56, 66]. However, in MS, as recently reported by Moore et al. [48], deposition of C9neo is observed predominantly in stromal concretions and at the subepithelial basement membrane, with only infrequent staining on choroid epithelial cells. Our study corroborates this finding: in Fig. 9j, we show that C9neo is almost entirely restricted to subepithelial basement membrane and stromal concretions in MS tissue. What sets NMO apart from the normal, non-pathogenic deposition of complement within the choroid stroma is the presence of AQP4 on the basolateral membrane of the epithelial cells adjacent to the basement membrane and stroma. Rather than accumulate in concretions, some of the circulating NMO IgG that crosses into the choroid stroma may find antigenic target and trigger complement deposition on the epithelial cell membranes. This is supported by the staining in Fig. 9d, in which C9neo is clearly present on the epithelial cell membrane. Thus, while immunoglobulin and complement deposition in the choroid plexus are not unique to NMO, per se, the distribution of these factors beyond the stroma and basement membrane is uniquely driven by the availability of the AQP4 target antigen and the presence of anti-AQP4 antibody in NMO patients.

The presence of C9neo on choroid epithelial cells raises the possibility of complement-mediated damage to the choroid as a pathogenic trigger in NMO. However, paradoxically, while we observed ependymal disorganization and discontinuity coincident with C9neo deposition and subpial vacuolations associated with C9neo deposition at the pial glia limitans, we did not find evidence for overt choroid plexus damage. In this context, it is noteworthy that AQP4 is also expressed on the basolateral membrane of kidney epithelial cells in the distal tubule [72] and is accessible to NMO IgG across the peritubular capillary endothelial cells in the same manner that it is accessible in the choroid plexus [36]. However, kidney pathology has not been widely reported in NMO patients, with only two studies suggesting possible injury to AQP4-expressing tubular epithelial cells, both of which occurred in unusual circumstances [25, 49]. This may be explained by the robust expression of complement regulatory factors on the basolateral membrane of distal tubule and collecting duct cells in the kidney [63]. Choroid plexus epithelial cells also express complement regulatory proteins and upregulate expression of factors, such as CD46 and CD59 under pathogenic conditions [5, 68]. Moreover, several studies indicate that C9neo deposition, despite being equated with obligate lytic destruction in much of the literature, can be detected in the absence of damage due to regulation of the terminal membrane attack complex by molecules, such as vitronectin and clusterin [7, 21]. The expression of these molecules in the human choroid plexus is currently unknown, but the maintenance of physical integrity at the blood–CSF barrier despite heightened C9neo deposition suggests that something is preventing lytic destruction. Likewise, while we speculate that the blood–CSF barrier may serve as a unique entry point for NMO IgG into the CNS, it is not clear whether such transmission requires a physical compromise of the barrier or whether changes in AQP4 expression, microglial activation, and complement cascade engagement initiate a functional compromise or modulation that permits IgG introduction from the blood into the CSF.

If circulating NMO IgG enters the CSF at the choroid plexus, it may initiate a cascade of events that eventually leads to parenchymal astrocyte injury and subsequent secondary loss of myelin [39]. Our observation of AQP4 expression on the basolateral surface of ependymal cells in normal tissue and the loss of ependymal AQP4 expression in NMO tissue, as well as evidence of ependymal discontinuity and C9neo deposition on ependymal cells, suggests that CSF NMO IgG gained access to this CSF–brain barrier and elicited damage. Likewise, astrocyte reactivity, loss of AQP4 expression, and deposition of C9neo at the pial glia limitans, associated with subpial vacuolated tissue, suggest that this CSF–brain barrier was also targeted by NMO IgG. While ependymal cells along the ventricle are connected by intermediate junctions [51] and pial fibroblasts are joined by desmosomes [18], neither of these structures are as restrictive to IgG diffusion as tight junctions [44]. At these sites, NMO IgG would have access to AQP4 expressed on the basolateral surface of ependymal cells as well as to AQP4 expressed on astrocytic endfeet. Thus, serum NMO IgG may gain access to brain parenchyma by transitioning through the blood–CSF barrier at the choroid plexus, circulating through the CSF, and then transitioning through the CSF–brain barriers at the ependyma and the pial glia limitans. Ultimately, this blood-to-CSF-to-brain transition of NMO IgG may result in an inside-out antibody-mediated compromise of the blood–brain barrier, providing direct access of NMO IgG from blood-to-brain. In addition, the CSF route for NMO IgG entry into the brain parenchyma could generate reactive astrocyte responses that drive granulocytic infiltration even across an intact blood–brain barrier [23, 77], inducing inflammatory-mediated, leukocyte-dependent pathology within the parenchyma [40].

Our findings, therefore, suggest a possible pathogenic role for NMO IgG in the CSF. NMO IgG is detected in CSF and levels are predicted by recent relapse and high serum NMO IgG titer [26, 43]. Increased total CSF protein and elevated CSF lactate and albumin levels also correlate with NMO disease severity and acute relapse, suggesting a relationship between dysfunction at the blood–CSF barrier and disability [26, 31, 82]. Conversely, in a study of ten NMO patients with CSF NMO IgG measured at relapse, follow-up measurements after treatment revealed that a reduction in NMO IgG in CSF correlated with clinical improvement, though serum levels were not correlated with remission [11]. The concept of distinct pathogenic roles for CSF and serum NMO IgG is further supported by evidence that plasmapheresis showed delayed efficacy in some NMO patients, suggesting that the initial washout was insufficient to improve function [30, 38]. Together, these findings support the CSF compartment as an important source of pathogenic NMO IgG. The cascades elicited by CSF NMO IgG likely include the direct induction of complement-mediated injury to CSF–brain interfaces and parenchymal astrocytes, as well as the initiation of self-amplifying astrocytic stress responses, as discussed above. However, an equally important mechanism of injury triggered by NMO IgG in the CSF may be disruption of normal water homeostasis at the blood–CSF and CSF–brain barriers.

Pathologic changes in brain water content manifest in two distinct but inter-related ways: edema, associated with increased water in the brain parenchyma [71], and hydrocephalus, associated with increased water in the CSF [8]. Edema is generally categorized as cytotoxic, in which reduced water transport function in parenchymal cells leads to interstitial shrinkage and intracellular swelling in the absence of blood–brain disruption, or vasogenic, in which blood–brain barrier damage induces interstitial accumulation of water. Brain edema is cleared by interstitial or cellular water flow across the glia limitans superficialis into CSF and then into the blood, by water flow across the ependyma into the CSF and then into the blood, and by flow across the glia limitans perivascularis directly into the blood [70]. AQP4 is clearly involved in edema [71], such that genetic deletion of AQP4 results in reduced cytotoxic edema by limiting the rate of water accumulation in the parenchyma, but increased vasogenic edema by reducing water clearance at the CSF–brain and blood–CSF barriers [62]. In NMO, this suggests that loss of AQP4-expressing cells or loss of AQP4 expression at the CSF–brain barrier in the ependyma or pial glia limitans will reduce the capacity to remove water from the parenchyma, facilitating edema. Indeed, evidence of extensive T2-weighted MRI hyperintensities in subcortical white matter coupled to an absence of gadolinium enhancement by T1 MRI and an increase in the apparent diffusion coefficient indicates the presence of pseudo-vasogenic interstitial edema in NMO [64]. Similar observations of increased apparent diffusion coefficient in normal-appearing white matter in the absence of gadolinium enhancement [27] further argue for a pseudo-vasogenic edema in NMO in which interstitial water is increased without blood–brain barrier dysfunction due to failure to transport water at CSF–brain interfaces [12]. This model is consistent with the loss of AQP4 expression and vacuolated underlying tissue observed at these barrier sites in NMO, and with our previous report of intramyelinic edema [20]. This model also highlights the role of AQP4 loss at CSF–brain interfaces in the transient edema we previously described in NMO patients with posterior reversible encephalopathy syndrome [42].

We recently reported that obstructive hydrocephalus is found in NMO patients at a higher frequency than observed in the general adult population [6]. On the basis of these cases, we predicted a primary involvement of ependyma in NMO and our current findings support this concept. However, the pathogenic mechanisms underlying hydrocephalus in NMO are not straightforward. While evidence from mice lacking AQP4 expression indicates about a 10% incidence of hydrocephalus triggered by complete obstruction of the cerebral aqueduct [14], it is not clear why such obstructions occur. AQP4-null mice exhibit an intact blood–brain barrier but a disorganized ependymal cell layer, even in animals without hydrocephalus [14], supporting a model in which loss of AQP4, whether genetic or antibody-mediated, induces defects in CSF homeostasis. However, loss of AQP4 at the blood–CSF barrier in the choroid plexus may have counteracting effects on CSF production, resulting in little change in overall CSF volume. This model argues against CSF hyper-production as the cause of hydrocephalus in NMO, though it remains possible that hypo-production of CSF due to loss of choroid plexus epithelial cells could impact ventricular flow in a manner that increases the likelihood of aqueductal blockage. Such blockage and subsequent obstructive hydrocephalus may also arise as a result of ependymal cell loss induced by the NMO IgG-mediated complement deposition we observed in this study. Humans and mice with primary ciliary dyskinesia exhibit hydrocephalus [32], and genetic deletion or mutation of multiple specific ciliary proteins results in ependymal cilia beat defects and consequent hydrocephalus induced by reduced CSF flow [15, 33, 45, 79]. Additional evidence indicates that laminar CSF propulsion mediated by ependymal cilia is required to maintain aqueductal patency, and defects in such propulsion result in aqueduct stenosis and triventricular hydrocephalus [24]. Ultimately, short of catastrophic loss of ependymal cells and/or choroid plexus epithelial cells or the initiation of an amplification loop that results in a rapid rise in intracerebral pressure and consequent collapse of the aqueduct, it is likely that overt hydrocephalus in NMO is at one end of a spectrum that more frequently manifests as transient pressure increases and disorganized CSF flow. Regardless, these events may contribute to pathologic events in the brain parenchyma that run in parallel to direct NMO IgG-induced astrocyte pathology. Indeed, it is possible that preventing damage at the blood–CSF and CSF–brain interfaces may substantially reduce or delay direct parenchymal damage by the NMO IgG. In light of this, it is noteworthy that we observed robust complement deposition at the choroid plexus and ependymal interfaces, suggesting that these sites may be directly protected by complement cascade blocking therapies, such as eculizumab [57]. It would be of interest in future studies to determine if eculizumab therapy alters the imaging hallmarks of pseudo-vasogenic edema in NMO patients as a secondary outcome measure.

The pathology findings we describe also provide mechanistic evidence for our previous report of periependymal and periventricular MRI abnormalities [58]. These included fluid-attenuated inversion recovery (FLAIR) and T2 signal abnormalities in periependymal regions of the lateral ventricles and along the walls of the third and fourth ventricles and the aqueduct of Sylvius. In meningitis, FLAIR hyperintensity is caused by elevated CSF protein content related to leptomeningeal inflammation [29], which extends the effective echo time beyond the inversion time used to suppress bulk CSF [47]. This same effect results in T2 hyperintensity. Therefore, our previously reported MRI findings are consistent with pathological changes at the ependymal CSF–brain and choroid plexus blood–CSF barriers that may involve both inflammation (Fig. 6k, l) and decreased local CSF flow due to cell injury or loss and barrier breach (Fig. 6i, j). Likewise, the reports of the so-called “pencil thin” ependymal hyperintensities with contrast-enhanced FLAIR imaging [2] support a dynamic alteration or disruption at the CSF–brain barrier. Notably, in common with infection-induced changes in the ependyma and choroid plexus [16], intraventricular exudate (“ventricular debris”) may be an expected MRI observation during acute attacks in NMO patients and may contribute to a loss of aqueduct patency that triggers hydrocephalus [60].

In conclusion, we provide evidence that the interfaces between blood and CSF and between CSF and brain are key sites for the initiation of NMO pathophysiology. Furthermore, the pathology at these sites may have important implications for disease evolution in NMO patients, including serving as the possible point at which serum NMO IgG enters the CNS and gains broad access to the brain parenchyma. Our findings also indicate that NMO is an “AQP4-opathy” with pathological targets beyond the astrocyte and provide insight into recent reports of edema [12, 20, 27, 42, 64] and hydrocephalus [6] in NMO patients that may suggest a unique model for CSF flow-dependent pathogenic events in this disease. Finally, the abundance of complement activation products in the choroid plexus, pial glia limitans, and ependymal layer suggests that these sites may be key targets for complement blocking therapies [57]. As such, imaging of these interfaces and other measures of CSF homeostasis may offer quantifiable tests of efficacy in future trials using therapies that target the complement pathway in NMO.

## Electronic supplementary material

Below is the link to the electronic supplementary material.

Supplementary material 1 (DOCX 6066 kb)
