# Supplementary material for: Pathogenic implications of cerebrospinal fluid barrier pathology in neuromyelitis optica
Source: Acta Neuropathol. 2017 Feb 9;133(4):597–612. doi: 10.1007/s00401-017-1682-1 (PMC5348570; doi:10.1007/s00401-017-1682-1)
Supplement: Supplementary file 1 — Supplementary material 1 (DOCX 6066 kb) [file 401_2017_1682_MOESM1_ESM.docx]

**Supplementary Figure 1. NMO case selection flowchart.**


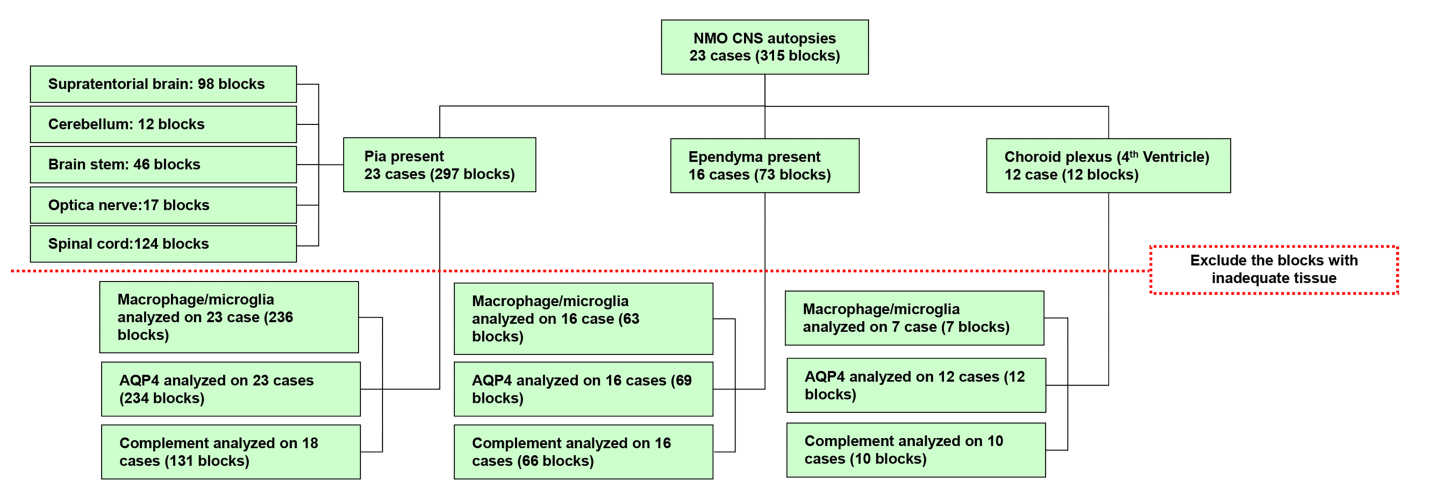


Schematic representation of the tissue analysis employed in this study.

**Supplementary Figure 2. GFAP immunoreactivity at the pial surface of the cerebellum in NMO and normal tissue.**

**
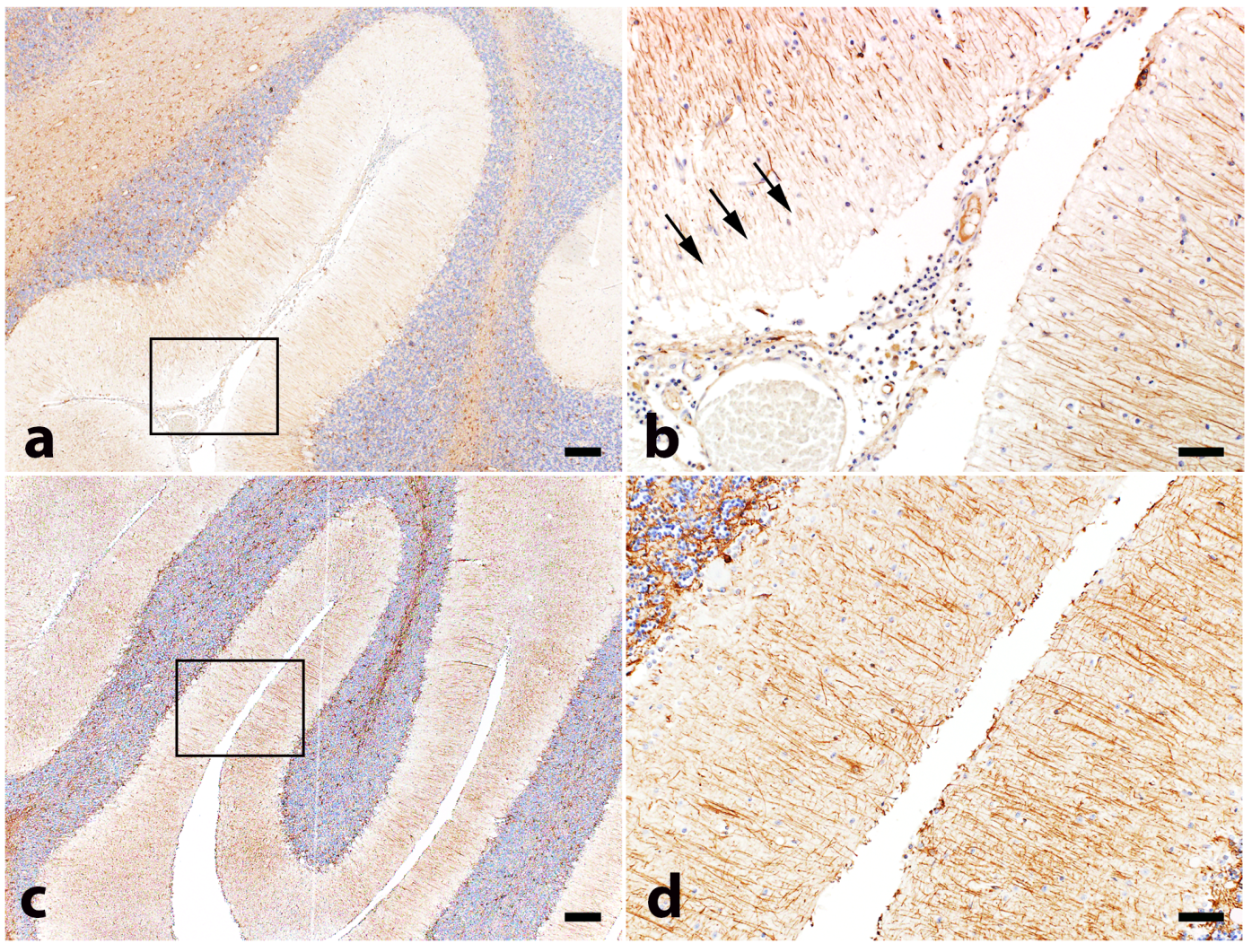
**

GFAP immunohistochemistry at the pial cerebellar surface in NMO demonstrates mild loss of astrocytic processes (a, higher magnification in b), compared to normal human cerebellum (c, higher magnification in d). Scale bars in a and c = 200 μm, scale bars in b and d = 50 μm.

**Supplementary Figure 3**. **Anatomical distribution of changes in pial AQP4 immunoreactivity in individual NMO patients.**


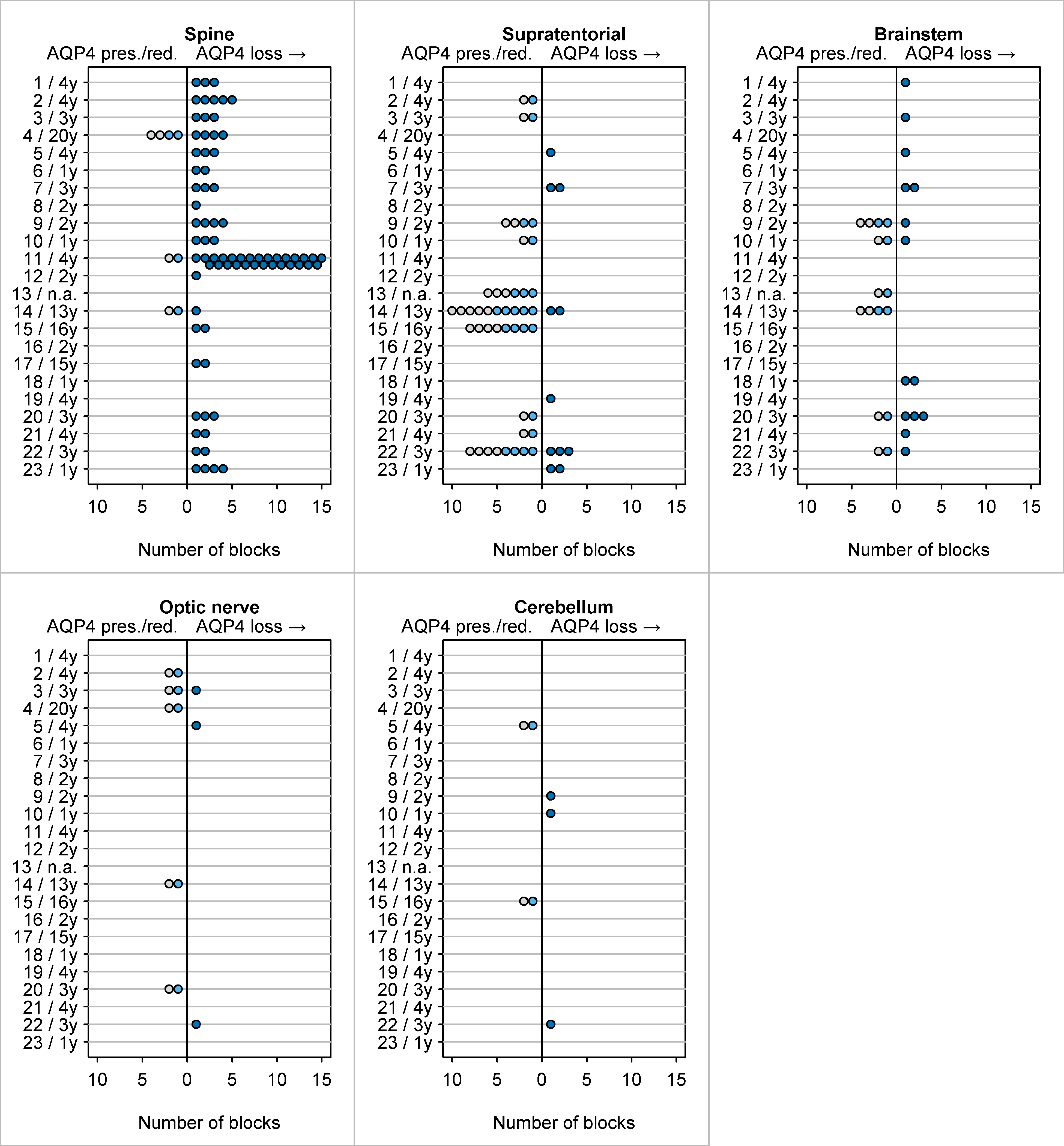


Patient identifiers and disease duration (years) are shown on the y-axis. Dark blue points to the right of the central vertical line enumerate blocks with AQP4 loss. Light blue points to the left of the vertical line enumerate blocks that showed decreased AQP4 but not loss. Gray points to the left of the vertical line enumerate blocks that had only normal or increased AQP4. One individual had more than 15 blocks with AQP4 loss and the points were wrapped onto a second line.

**Supplementary Figure 4.** **The pattern of microglial reactivity in the pia in individual NMO patients.**


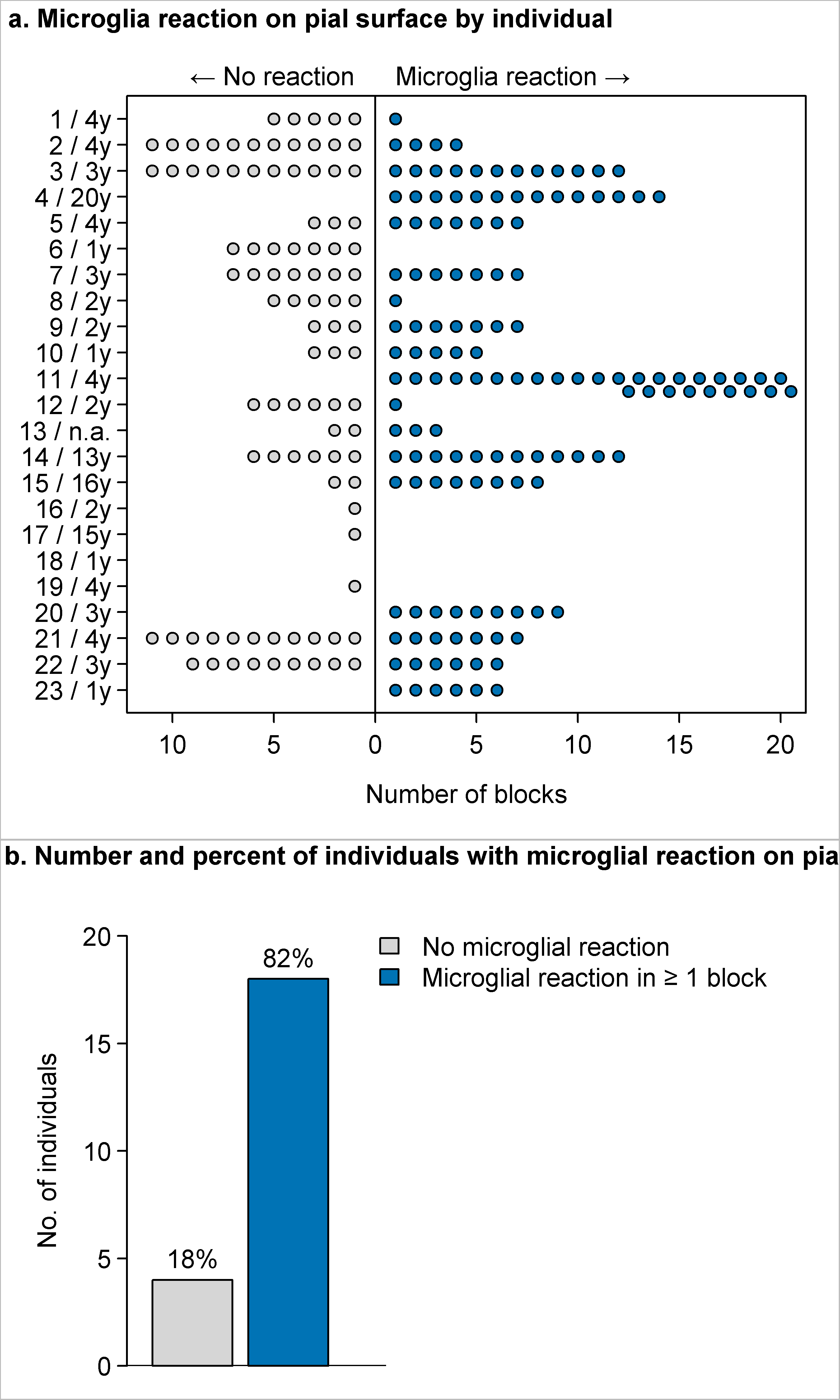


Patient identifiers and disease duration (years) are shown on the y-axis (a). Dark blue points to the right of the central vertical line enumerate blocks with pial microglial reactivity. Gray points to the left of the vertical line enumerate blocks that didn’t show pial microglial reactivity. b) 78% of the available NMO patient tissues exhibited microglial reactivity in the pia in at least one block.

**Supplementary Figure 5.** **Pattern of C9neo deposition in the pia in individual NMO patients.**


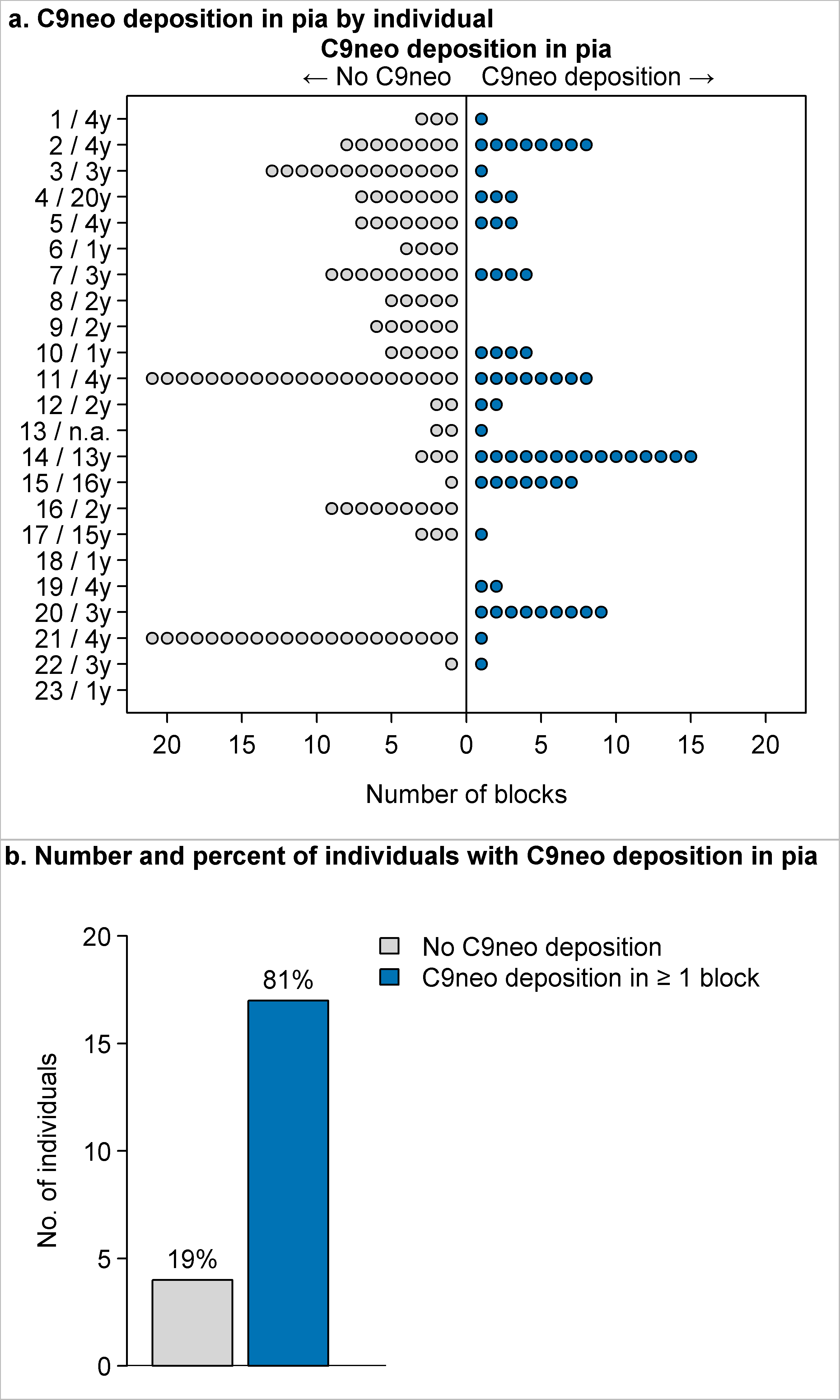


Patient identifiers and disease duration (years) are shown on the y-axis (a). Dark blue points to the right of the central vertical line enumerate blocks with C9neo deposition. Gray points to the left of the vertical line enumerate blocks that had no C9neo deposition. b) 74% of the available NMO patient tissues exhibited C9neo deposition in the pia in at least one block.

**Supplementary Figure 6. Subependymal GFAP immunoreactivity in NMO.**

**
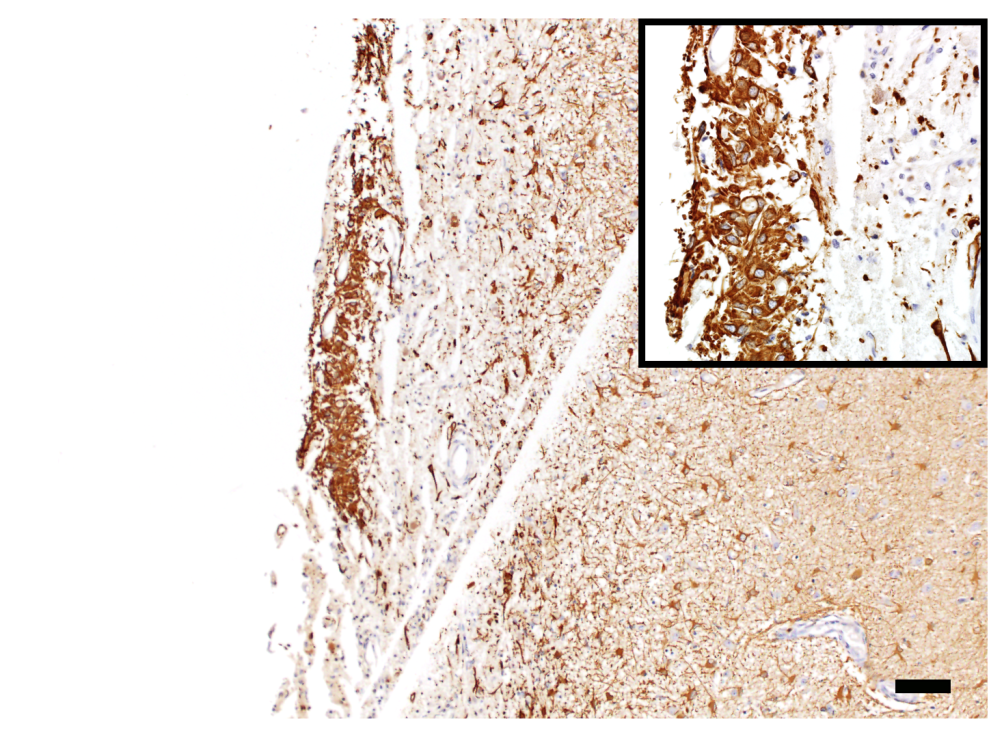
**

GFAP immunostaining shows both reactive and damaged astrocytes in the subependymal region of tissue from an NMO patient (complementary to Figure 6o). Scale bar=20 µm.

**Supplementary Figure 7.** **The pattern of microglial reactivity in the ependyma in individual NMO patients.**


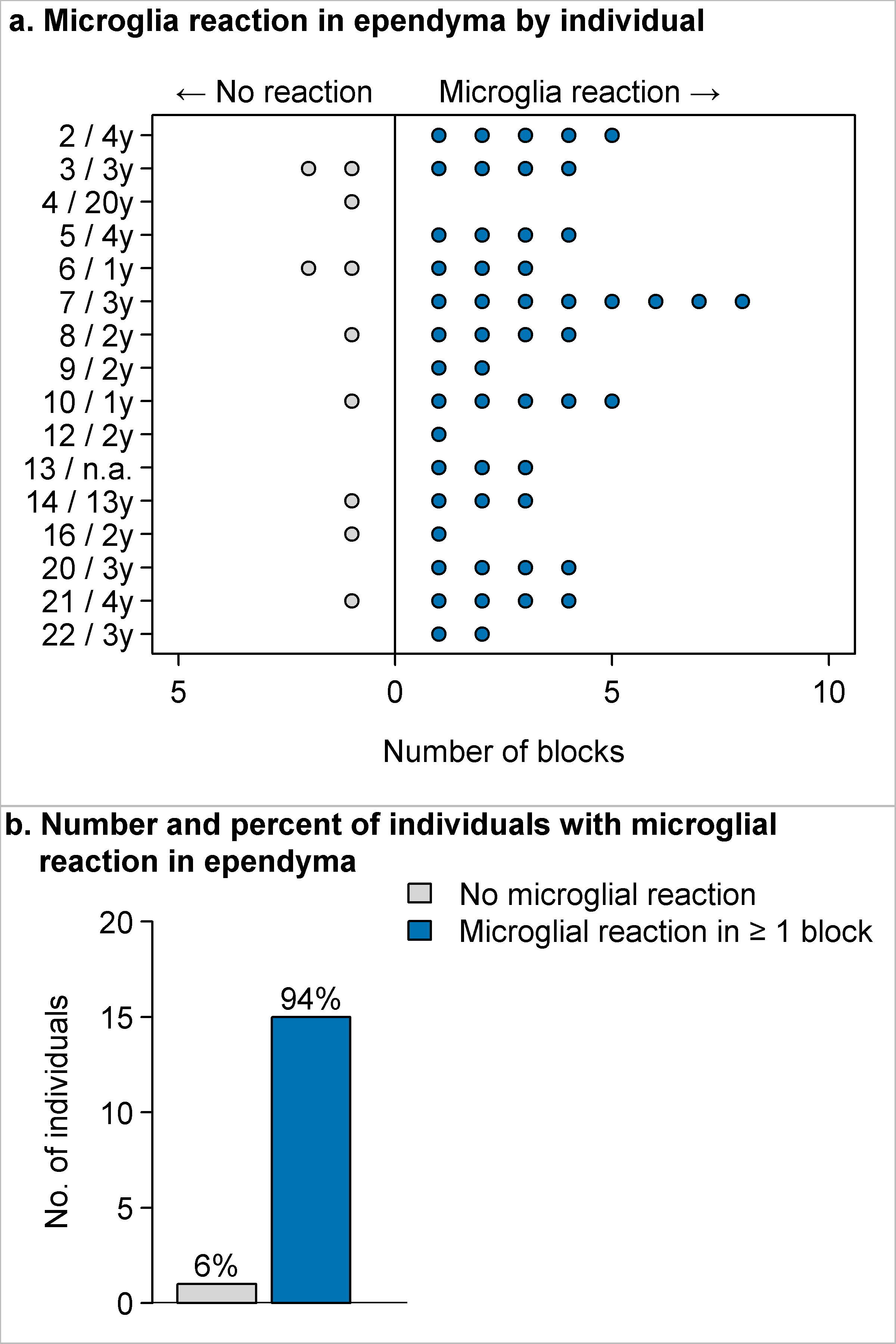


Patient identifiers and disease duration (years) are shown on the y-axis (a). Dark blue points to the right of the central vertical line enumerate blocks with microglial reactivity in the ependyma. Gray points to the left of the vertical line enumerate blocks which didn’t show ependymal microglial reactivity. b) 94% of the available NMO patient tissues exhibited ependymal microglial reactivity in at least one block.

**Supplementary Figure 8.** **The pattern of C9neo deposition in the ependyma in individual NMO patients.**


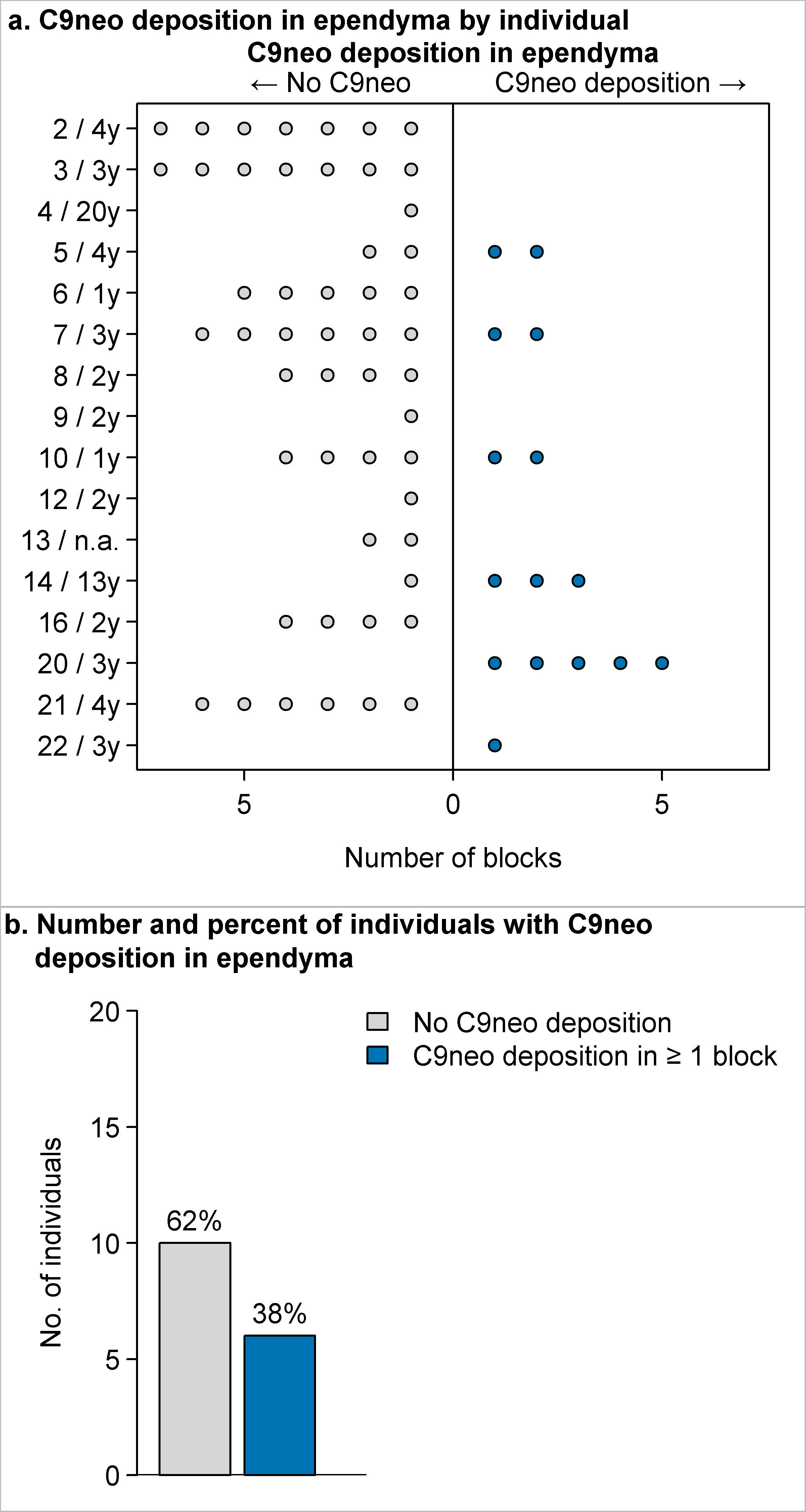


Patient identifiers and disease duration (years) are shown on the y-axis (a). Dark blue points to the right of the central vertical line enumerate blocks with C9neo deposition. Gray points to the left of the vertical line enumerate blocks that had no C9neo deposition. b) 38% of the available NMO patient tissues exhibited C9neo deposition in the ependyma in at least one block.
